# A quantitative assessment of the Hadoop framework for analyzing massively parallel DNA sequencing data

**DOI:** 10.1186/s13742-015-0058-5

**Published:** 2015-06-04

**Authors:** Alexey Siretskiy, Tore Sundqvist, Mikhail Voznesenskiy, Ola Spjuth

**Affiliations:** 1 Department of Information Technology, Uppsala University, P.O. Box 337, Uppsala, SE-75105 Sweden; 2 Department of Physical Chemistry, institute of Chemistry, St-Petersburg State University, Saint-Petersburg, Russia; 3 Department of Pharmaceutical Biosciences and Science for Life Laboratory, Uppsala University, P.O. Box 541, Uppsala, SE-75124 Sweden

**Keywords:** Next generation sequencing, Massively parallel sequencing, Hadoop, High-performance computing, DNA-seq, Bioinformatics

## Abstract

**Background:**

New high-throughput technologies, such as massively parallel sequencing, have transformed the life sciences into a data-intensive field. The most common e-infrastructure for analyzing this data consists of batch systems that are based on high-performance computing resources; however, the bioinformatics software that is built on this platform does not scale well in the general case. Recently, the Hadoop platform has emerged as an interesting option to address the challenges of increasingly large datasets with distributed storage, distributed processing, built-in data locality, fault tolerance, and an appealing programming methodology.

**Results:**

In this work we introduce metrics and report on a quantitative comparison between Hadoop and a single node of conventional high-performance computing resources for the tasks of short read mapping and variant calling. We calculate efficiency as a function of data size and observe that the Hadoop platform is more efficient for biologically relevant data sizes in terms of computing hours for both split and un-split data files. We also quantify the advantages of the data locality provided by Hadoop for NGS problems, and show that a classical architecture with network-attached storage will not scale when computing resources increase in numbers. Measurements were performed using ten datasets of different sizes, up to 100 gigabases, using the pipeline implemented in Crossbow. To make a fair comparison, we implemented an improved preprocessor for Hadoop with better performance for splittable data files. For improved usability, we implemented a graphical user interface for Crossbow in a private cloud environment using the CloudGene platform. All of the code and data in this study are freely available as open source in public repositories.

**Conclusions:**

From our experiments we can conclude that the improved Hadoop pipeline scales better than the same pipeline on high-performance computing resources, we also conclude that Hadoop is an economically viable option for the common data sizes that are currently used in massively parallel sequencing. Given that datasets are expected to increase over time, Hadoop is a framework that we envision will have an increasingly important role in future biological data analysis.

**Electronic supplementary material:**

The online version of this article (doi:10.1186/s13742-015-0058-5) contains supplementary material, which is available to authorized users.

## Background

Since its inception, massively parallel DNA sequencing, also referred to as Next Generation Sequencing (NGS), has been an extremely bountiful source of data, providing insights into the workings of biological machinery [1,2]. It decreases the costs of sequencing as well as facilitates and promotes bigger studies with increasingly larger dataset sizes. Extracting useful information from these voluminous amounts of data is transforming biology into a data-intensive discipline requiring high-performance computing (HPC) infrastructures. As an example of the scale of the demands, a single Illumina high-throughput sequencing run produces approximately 1800 gigabases (Gb), corresponding to 2 terabytes (TB) of raw data, in 3 days [3].

Mapping short reads to a reference sequence and then finding the genetic variations specific to the sample is a common task in NGS data analysis. Many of the most widespread tools for this purpose (such as BWA [4], Bowtie [5], and Samtools [6]) are not designed with distributed computing in mind. Many others do not even have the native ability to use multiple cores.

Computers today consists of one or more processors, in turn containing one or more compute cores. In HPC, computers used for calculation are commonly referred to as computer nodes or simply nodes. The most widespread approach to accelerate NGS tools is to parallelize within a node (multi-core parallelization) using shared memory parallelism, such as with OpenMP [7], but this approach is naturally limited by the number of cores per node, which currently does not usually exceed 16 [8]. For the tools that do not support OpenMP natively (e.g. Samtools) variant calling can be parallelized by creating a separate process for each chromosome, genetic interval, or using the GNU Parallel Linux utility [9]. It is important to note that a multi-core approach does not improve the performance of operations that are limited by local disk or network throughputs, whereas splitting the dataset and using multi-node parallelization is not generally bound by these constraints. Message Passing Interface (MPI) is a common method to implement multi-node parallelization [10]. However, writing efficient MPI programs for hundreds of cores is a non-trivial task because thread synchronization (or load balancing) has to be woven into the software code and there are only a few existing solutions available for processing sequencing data [11,12].

Another approach to introduce parallelization into NGS analysis pipelines in Linux systems is to use custom scripting in a language such as Bash, Perl, or Python. This involves using existing utilities and cluster tools to split the data into chunks, process them on separate nodes, and merge the results afterwards. This kind of scatter-gather solution can also benefit from specialized implementation strategies based on MPI-like and OpenMP parallelization to provide good performance. However, this development requires substantial expertise to be efficient. Furthermore, given that the process is tightly coupled to the local computational cluster and network architectures, it might also be nearly impossible to exploit such optimizations in different settings.

Other examples of strategies for parallelization of NGS analysis includes the Galaxy platform [13], which provides a built-in parallelism functionality that automatically splits the input dataset into many part files on the shared file system, uses the cluster to process them in parallel, and then collects the results. However, when it is used as a data communication medium to preserve good scalability, the shared file system puts essential demands on the internode communication network infrastructure. Another factor limiting scalability is the system’s inability to cope with transient system problems and failed cluster nodes, either of which would simply result in a failed job. The GATK’s Queue pipeline tool [14] provides a more advanced solution that deals with this latter scalability problem but not the former because it also uses a shared file system to move data betweennodes.

Unlike these strategies, which tend to wrap existing tools in a parallelization scheme, a more radical option is to adopt the Map-Reduce (MR) programming model, which has been conceived with the objective of achieving scalability [15]. MR offers a compelling alternative for running tasks in a massively parallel way and it is successfully used by many well-known data-driven operations [16] to process huge datasets with sizes of up to terabytes [17] and even petabytes [18]. The most prevalent open source implementation of MR is Hadoop [19]. The Hadoop MR framework has an appealing programming methodology in which programmers mainly need to implement two functions: *map* (mapper) and *reduce* (reducer). The framework provides automatic distribution of computations over many nodes as well as automatic failure recovery (by retrying failed tasks on different nodes). It also offers automated collection of results [19]. The Hadoop Distributed File System (HDFS) is a complementary component that stores data by automatically distributing it over the entire cluster, writing data blocks onto the local disk of each node and, therefore, effectively enables moving the computation to the data and thus reduces network traffic. HDFS provides a storage system where the bandwidth and size scales with the number of nodes in the cluster [20], which is very different from the properties of the usual HPC cluster networkarchitecture.

There are currently several Hadoop-based solutions that can be used to analyze the results for DNA-seq [21,22], RNA-seq [23], and de-novo assembly [24] experiments; see also [25] for an overview. Although previous reports, such as the Crossbow publication [22], have mainly focused on the performance of Hadoop tools on public clouds and, in particular, Amazon EC2, a thorough comparison of similar analysis pipelines on an HPC cluster and a private Hadoop cluster is notavailable.

In this manuscript we focus on quantifying the common belief that Hadoop excels at analyzing large NGS datasets and we study the execution times for datasets of different sizes. We also compare the Hadoop and HPC approaches from a scaling perspective.

## Data description

Previously published publicly available datasets are used, see Methods section for more.

## Methods

### Datasets

For our experiments we used a publicly available Dataset I to check the concordance of the HPC and Hadoop approaches and a synthetic Dataset (S) that was generated from the *Arabidopsis thaliana* TAIR10 [26] reference genome with the wgsim tool from the Samtools package, which mimicked Illumina HiSeq sequencing data. The datasets are listed in Table 1.

**Table 1:** **Datasets used in the comparison**

Dataset	Organism	Size in Gb
I	*A.thaliana*	1.4
S	*A.thaliana*, the artificial dataset	100.0
	created using Samtools package	

### Data preparation

We derived nine datasets of pair-ended reads by extracting a progressively increasing part of Dataset S, uniformly occupying the range from approximately 10 to 100 Gb, which are referred to as Datasets S1-S9. All of the data used were standardized to the FASTQ format and compressed with pbzip2 [27] (which is a parallel version of the bzip2) because it provides very good compression and is splittable. This means that the archive can be natively expanded by Hadoop using the proper codecs in a massively parallel manner, thereby allowing us to simultaneously process multiple segments [19,20].

The indexed reference genome was copied to Hadoop’s file system (HDFS) before starting the experiments. Specifically, the storage volume containing the input data was mounted with SSHFS to one of the Hadoop nodes, from where the bzip2-compressed data was transferred to HDFS.

### Computational resources

We used a computational cluster at UPPMAX [28] to run the HPC analysis pipeline. Data and reference genomes were stored on a parallel, shared storage system. The Hadoop test platform was deployed on a private cloud using the OpenNebula [29] cloud manager and it was configured with the Cloudera Hadoop distribution version 2.0.0-mr1-cdh4.7.0 [30].

Each node in the HPC cluster is equipped with 16 cores and 24-96 GB RAM, while each node in the private cloud has seven cores and 62 GB of RAM (referred to as Hadoop I). The underlying hardware for the Hadoop I cluster allows for 8.8 GB of RAM per core of the virtual nodes enabled by the KVM hypervisor [31]. Having this amount of RAM makes it feasible to enable hyper-threading; that is, for each physical core on the physical node, the host operating system addresses two logical cores, which increases the number of CPU cores on the physical node to 16. In this case, one virtual node has 14 cores with about 4 GB RAM per core, which allows us to run the Hadoop services while keeping in memory a reference genome as large as the *Homo sapiens* indexed by Bowtie. We refer to the hyper-threaded version as the Hadoop II cluster. A detailed specification of the hardware configuration of the two computational resources is available in the Additional file 1.

### Analysis pipelines

To compare the Hadoop and HPC platforms, we chose the task of identifying single-nucleotide polymorphisms (SNPs) from short-read data. Although there are many different approaches and software tools to perform this task in an HPC environment [32], there are only a limited number of options for Hadoop.

In an attempt to make a fair comparison between the two very different platforms, the Crossbow package [22] (version 1.2.0), which performs the tasks of preprocessing, mapping, and variant calling from short reads, was chosen. This package may be run in both Hadoop and HPC environments, invoking the same mapper (Bowtie [5], version 0.12.8) and SNP caller (SoapSNP [33], version 1.02). We constructed two separate pipelines: the first runs on HPC resources and the second runs on a Hadoop system. We introduced the changes to Crossbow that are described in Section Data preprocessing, which enables us to preprocess the reads in a massively parallel way.

We designed an additional experiment to study the advantages of the data locality strategy used by Hadoop in comparison to the NAS or SAN solutions that are widely used in HPC centers. On the NAS storage area with enabled data striping, we used Dataset S to map it to the reference genome TAIR10 with the following strategies:

1. The dataset from the NAS was copied in a *parallel* way to a set of HPC computation nodes, so that the local disk of each node received a chunk of the initial dataset, which was continued with the local data mapping with Bowtie, and ended with the mapped reads in the SAM format being sent back to the NAS.
2. The same dataset from the NAS was copied to HDFS, where the mapping to the same reference genome with the same mapper was carried out and the mapped data was left on the HDFS.

We measured the run time for each of the pipelines, distinguishing the communication time and mapping time. For the HPC nodes, communication is a sum of the time for copying the data to and from computation nodes, while for Hadoop the sum of data ingesting and preprocessing times was counted as the communication expense. The exact workflow for HPC is available fromGithub [34].

## Results

### Data preprocessing

Crossbow includes a preprocessor that can download data from Amazon S3, FTP, and HTTP servers over the internet, which it then reshapes and delivers to the Hadoop cluster [22]. To provide the URLs of the data, Crossbow uses a manifest file where each line describes a location of one dataset. The preprocessor launches one mapper per line to split and reformat the data. Therefore, the only way to parallelize the preprocessing stage is to manually split the dataset into subsets and list their locations in the manifest file.

We have developed a massively parallel Python implementation of the preprocessor [35], making use of the Hadoop Streaming library. The preprocessor can process either different versions of FASTQ-formatted data from the Illumina platform or data extracted from the SRA format [36]. Our solution imposes the requirement that the input data is stored where the Hadoop nodes can access it (for example, on HDFS) and is compressed in a splittable archive (such as bz2). To improve memory efficiency in a Hadoop setting, we introduced a standard for the FASTQ read IDs by generating new IDs based on the SHA-1 hash function, we then labeled the read mates with “.1” and “.2” suffixes. The benefit of this is that by knowing the exact size of the read IDs for the whole dataset, we can avoid unnecessary memory spills in the Hadoop implementation during the sorting stage.

#### Re-archiving the data

It is common for sequencing platforms to deliver data in the gz format. However, despite its convenience, it cannot be unpacked in parallel, which is contrast to the bz2 format that is utilized by our short read preprocessor. We measured the time for (gzip-bzip2) conversion versus (gzip-split-gzip) for different dataset sizes using the highly efficient GNU parallel tool and the pigz tool [37] on a multi-core node. For the datasets, we observed that timings for the bzip2 compression are smaller, while the data is stored more efficiently (see Table 2).

**Table 2:** **Timings in seconds for the different pipeline stages when running Crossbow on HPC node (16 CPU cores) and Hadoop I cluster (eight nodes, 56 CPU cores) and Hadoop II cluster (eight nodes, 112 CPU cores) for Datasets S1-S9**

Stages	Platform	Datasets								
		S1	S2	S3	S4	S5	S6	S7	S8	S9
ingest to HDFS	Hadoop I,II	106	236	472	606	862	974	1018	1244	1384
conversion	gz split (HPC)	466	782	1094	1406	1728	2052	2390	2774	3090
	gz to bz2 conversion (Hadoop I,II)	211	423	633	842	1056	1264	1473	1685	1911
preprocess	HPC	406	630	1002	1235	1469	1810	2043	2283	2660
	Hadoop I	560	891	1172	1672	1937	2271	2665	3011	3396
	Hadoop II	537	685	892	1179	1414	1641	2091	2334	2613
map	HPC	1434	2857	4281	5775	7216	8627	10088	11432	13028
	Hadoop I	707	1385	2060	3331	3398	4163	4761	5630	6276
	Hadoop II	511	981	1459	2636	3023	3194	3361	4553	4766
	Hadoop II*	486	955	1422	1882	2336	2812	3310	3771	4305
SNP call	HPC	1045	1698	2621	3553	10989	18993	16890	20785	21948
	Hadoop I	666	994	1127	1423	1906	2287	2765	2982	3444
	Hadoop II	661	965	1344	1364	1830	2450	2765	3029	3471
total time	HPC	3351	5967	8998	11968	21402	31554	31411	37274	40726
	Hadoop I	2250	3929	5464	7848	9159	10959	12682	14558	16436
	Hadoop II	2026	3289	4601	6607	7719	8903	10393	12845	14145

The ‘Hadoop II*’ data were obtained as follows: the average time for each mapping job was multiplied by the number of successful Hadoop mapping jobs, omitting the failed jobs. The errors are not shown.

#### Efficiency of the preprocessing stage

**Figure 1:**
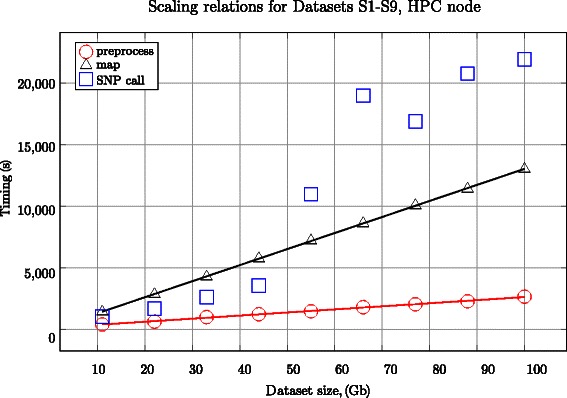
Scaling relations for different stages of the pipeline executed on the HPC node. Good data partitioning in the manifest file yields a linearscaling for the preprocessing stage. However, the SNP calling stage does not scale linearly, which is the main reason of the sub-optimal load balancing implemented in Crossbow.

**Figure 2:**
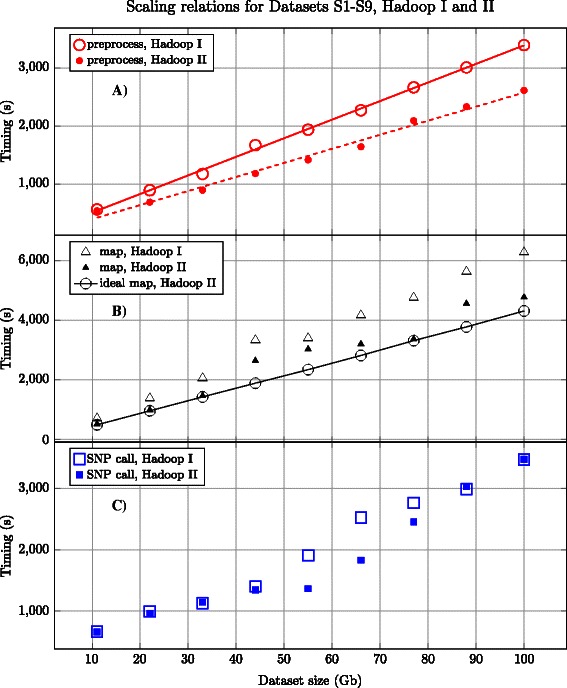
Scaling relations for Hadoop I and II with increasing input dataset size, from S1 to S9. The figure highlights the almost linear scaling characteristics of the Hadoop approach. Our MR implementation of the preprocessing stage **(A)** displays almost perfect linear scaling. The ‘ideal map’ curve for Hadoop II cluster (open circles) shows **(B)** the deviations from the real case (closed triangles) due to map jobs failures; that is, additional time to reallocate and restart jobs. The SNP calling stage **(C)** scales worst, compared with the others, mainly because of the poor job balancing of the Crossbow.

To improve the native Crossbow preprocessor scalability, all of the Datasets S1-S9 were split beforehand and stored on conventional HPC storage. The number of gzip splits varied from 23 for S1 to 201 for S9, providing a good load balancing for the 16 CPU core node. To show the scalability properties of our MR preprocessor, we measured its execution time for Datasets S1-S9 located on HDFS. The timings, which are included in Table 2, indicate that the scaling relations are close to linear in both cases, see Figures 1, 2. In the case of NGS data delivery in bz2 format, our approach is more advantageous because the data is ready for massively parallel processing. One of the benefits of the native preprocessor that should be raised is its great flexibility in downloading data from different sources.

### Mapping and variant calling

We executed the two pipelines for each of the nine datasets on the HPC node and Hadoop platforms, and measured the wall-clock run time for each pipeline stage. All of the experiments were repeated at least three times and the results were averaged to obtain a data point for the particular combination of data size and computational platform.

#### Concordance of Hadoop and HPC results

To check the agreement between the Hadoop and HPC pipelines, the produced SNP lists were compared for each of the platforms for Dataset I [38]. Of the total 39,129,856 reads that were processed with Bowtie, 23,014,231 reads with at least one reported alignment (i.e. 59% of mapped reads, which is not an unusual case for *A.thaliana*) were parsed by SOAPsnp to detect SNPs. From the total 100,162 SNPs detected by SOAPsnp, there were 212 records with minor differences, such as ‘Count of uniquely mapped second best base’, ‘Count of uniquely mapped best base’ etc. (see the SOAPsnp manual). The overlap between the SNP lists, taking into account the average sequencing depth of 10, indicates the practical equivalence of the pipelines. The short read length for the test Dataset I (only 30 bp), and the low average coverage and non-uniform distribution of the mapped reads along the genome, are responsible for producing the observed minor difference in the SNPs. If the average coverage increases, then the difference between the pipelines would disappear.

#### Scalability

Given the wide range of dataset sizes processed in practice (e.g. exomes and whole-genome sequencing at various depths) it is important to understand the scaling characteristics of the Hadoop and HPC processing platforms with respect to input size. We measured the time required to execute each platform’s respective analysis pipeline for each of the test Datasets S1-S9. The results are shown in Table 2 and Figures 1, 2, including the scaling relations for the pipeline stages. We observe that the preprocessing and mapping stages with Crossbow scale linearly with the dataset size. However, the SNP calling stage displays worse scalability for dataset sizes greater than 50 Gb. The deviation is due to Crossbow task scheduling, which results in a sub-optimal load balancing of the CPU cores. One could speculate that one of the reasons for this is an imperfect pileup strategy of the genomic regions with a highly divergent coverage by the mapped reads. The measurements for the Hadoop platform show perfect scaling for our MR preprocessor with respect to dataset size.

**Figure 3:**
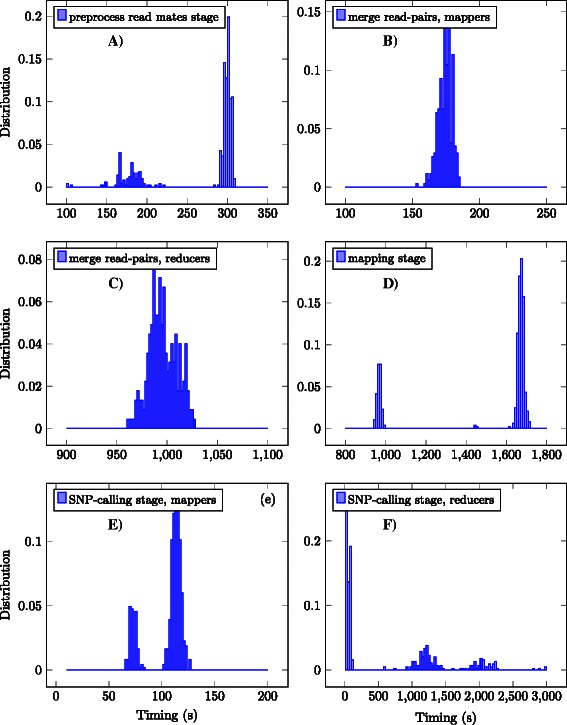
Timing histograms for different stages of the Hadoop-based Crossbow pipeline: preprocessing **(A)**-**(C)**, mapping **(D)** and SNP calling **(E)**-**(F)** for Hadoop II cluster, Dataset S9, several independent runs. The double maxima on some plots are due to round-robin-like fashion Hadoop distributes jobs among the worker nodes: the first full-size jobs are scheduled, while the remaining data chunks form smaller peaks of shorter duration. The stage tends to have better scaling when the double peaks, if any, are closer. From this perspective, the reason of sub-linear scaling of the Crossbow SNP-calling stage becomes clear: the majority of jobs of the reducing phase **(F)** are very short, in the order of tens of seconds, while there are several much longer jobs.

To estimate deviations from the linear scaling for the mapping stage, we calculated the average Hadoop job time multiplied by the number of successful Hadoop jobs for each Dataset S1-S9. The data is plotted as an ‘ideal map’ curve for each of Datasets S1-S9 in Figure 2 for the Hadoop II cluster, and are marked as ‘Hadoop II*’ in the Table 2. After a thorough analysis of the log files, we conclude that one of the reasons for the deviation is the failure of the Hadoop jobs, which results in job migration and restart. To enforce the statement, the statistics for the Hadoop II jobs processing the Dataset (S) were collected so as to plot the timing distributions for different stages of the Hadoop pipeline (Figure 3). The double maxima on Figure 3(A-E) are due to the round-robinpattern by which the jobs are distributed between the nodes: on the first round the full-size jobs are pushed, whereas the remaining dataset chunks of reduced size are processed faster, building up the left maximum. The distribution for the SNP-calling stage (Figure 3(F)) is different: most of the jobs have a duration in the order of seconds, while there are other jobs that run for almost one hour. These outliers contribute to the sub-linear scaling of the SNP-calling stage. We also note that enabling hyper-threading (Hadoop II) improves the performance. Despite the observed deviations from the ideal scaling, the Hadoop framework can help to engage a multitude of nodes, even if the underlying program does not scale beyond a single node [22,39].

#### Efficiency of the calculations

**Figure 4:**
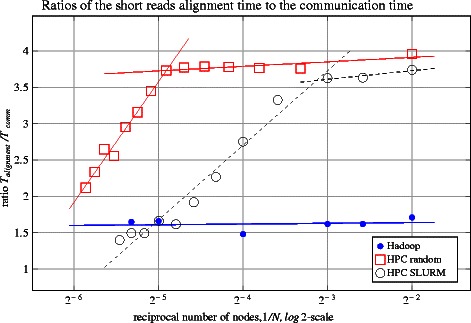
The ratio of the *F*_*Hadoop*_/*F*_*HPC*_ as a function of the reciprocal dataset size in Gb. The pipelines were run on the Hadoop I and II clusters, as well as a 16 core HPC node. The analytical curve *f*(*x*)=(*a*_1_ *x*+*b*_1_)/(*a*_2_ *x*+*b*_2_) was used to fit the data for the stretches of linear scaling of calculation time on the HPC platform. The outliers are marked with crossed symbols.

Given that Hadoop was designed to digest huge datasets [17,40], we hypothesize in terms of the total CPU time that the Hadoop platform becomes more efficient when compared with the HPC platform when the biological sequence dataset is larger. For comparison, we use the function *F*=*T*_*p*_×*p*, where *T*_*p*_ is the calculation time on *p* cores. To observe the relative scalability of the approaches, we introduce the *F*_*Hadoop*_/*F*_*HPC*_ metric and investigate its behavior for different dataset sizes. Importantly, the choice of metric makes it possible to compare the efficiencies of two very different platforms as a function of the dataset size, as shown in Figure 4, which displays the ratio *F*_*Hadoop*_/*F*_*HPC*_ for Hadoops I and II as a function of the *reciprocal* dataset size using the data from Table 2. Extrapolation to zero on the *X*-axis estimates the ratio for the hypothetical infinite dataset. For those stretches where both Hadoop and HPC display linear scaling, the analytical function *f*(*x*)=(*a*_1_*x*+*b*_1_)/(*a*_2_*x*+*b*_2_) was used to fit the points. The extrapolation for our settings gives the ratio as *F*_*Hadoop*_/*F*_*HPC*_≈1.4 for Hadoop I and ≈1.0 for Hadoop II. These asymptotes highlight that Hadoop can decrease the total run-time and, therefore, improve throughput by increasing the number of engaged cores, which is an option that is often not available for the HPC environment.

We note that the Hadoop platform becomes increasingly efficient compared to the HPC platform as the dataset size increases. The main reason for this is the sub-linear scaling of the Crossbow SNP calling phase on the HPC platform (Figure 1). This means that Hadoop, even when running in the virtualized environment of a private cloud assembled on moderate hardware, is competitive with the HPC approach run on ‘bare metal’ of relatively modern hardware without internode communication losses for datasets larger than 100 GB (Dataset S), which is typical for human genome sequencing with a sequencing depth ofabout 30*X*.

### Advantages of the data locality strategy

The network communication model for Hadoop has a major difference from the usual HPC cluster network architecture (*n*-tier tree) with NAS or NFS attached storages in that the effective bandwidth of the Hadoop network increases with the cluster size [20], which is in contrast to that of the HPC network where cluster growth results in network saturation and performance degradation. To study this phenomenon, we compared HPC and Hadoop network communication costs as a function of the number of nodes involved in a computation for Dataset S (100 Gb). Because the mapping process is embarrassingly parallel (i.e. the read-pairs are independent of each other and thus can be aligned independently) it is possible to distribute the work over many nodes (e.g. by splitting the initial data into chunks to process them in parallel). In this approach, reducing the size of each data chunk reduces the size of the mapper job, *T*_*alignment*_, but at the same time more chunks have to be simultaneously sent over the network, which increases the communication time, *T*_*comm*_.

There are several software packages for short-read mapping with MPI support [11,12]. Almost linear scaling up to 32 nodes for pair-ended reads has been reported with the Pmap package, which we observed to perform poorly for datasets larger than 20 Gb. Instead, we implemented a highly optimized Bash script to transfer FASTQ data from the NAS storage to the local nodes’ scratch disks, which were then aligned, and the SAM files were sent back to the NAS storage. This script is based on standard Unix utilities that make very efficient use of the HPC cluster network (the source code is available on Github [34]). We then compared the network performance with the standard Hadoop HDFSapproach.

**Figure 5:**
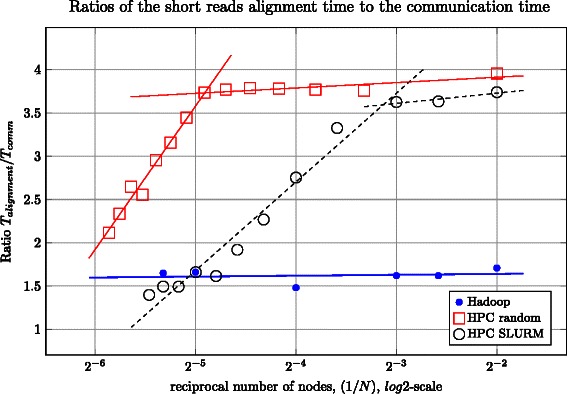
Ratios of the short reads alignment time to the communication time. All of the runs use Dataset S as an input. Two HPC scenarios are shown as ‘HPC SLURM’ and ‘HPC random’, which correspond to standard SLURM behavior and a modified behavior that allocates nodes from random racks. Linear fit was done with the least-squares method. One can see two defined linear regions for the HPC approach with very different tangents, which are caused by the network saturation. For the HPC platform, as the number of nodes increases a greater amount of time is spent on communication than on mapping. The Hadoop approach reveals better scaling properties.

We separated and calculated the arithmetic averages of the mapping time, *T*_*alignment*_, and communication time, *T*_*comm*_, and plotted the ratio *T*_*alignment*_/*T*_*comm*_ as a function of the reciprocal number of nodes 1/*N* involved in computations (Figure 5). Although this measure is applicable to both HPC and Hadoop, *T*_*comm*_ has a different interpretation for each of these platforms. For Hadoop, *T*_*comm*_ is the time needed to deliver the data to HDFS and preprocess the short reads in FASTQ format to prepare them for the core MR pipeline phases. In this scenario, a good data locality is automatically ensured by the distributed data storage strategy used by HDFS. For the HPC approach, *T*_*comm*_ is the time needed for the chunks to be sent from the data storage location to the node’s scratch disks (where the actual mapping happens) and to transfer the aligned SAM files back to the storage location over the network.

**Hadoop I results (filled circles in Figure**5**):** The ratio *T*_*alignment*_/*T*_*comm*_ indicates weak dependency in a wide range of number of nodes *N*: from 4 up to 40. It is known that Bowtie provides almost linear [5] scaling between mapping time and dataset chunk size *D*: *T*_*alignment*_∝*D*∝1/*N*. Because the ratio *T*_*alignment*_/*T*_*comm*_ is approximately constant, one can conclude that *T*_*comm*_∝1/*N*; that is, the data is processed more quickly when more nodes are involved.

**HPC results (open shapes in Figure**5**):**The data from the delivery location is split into chunks in parallel, which are simultaneously pushed to the local scratch disks of the HPC nodes allocated by SLURM [41]. One can see two distinct linear stretches: one stretch is for the range from 4 to about 12 nodes, and the other is from 12 up to 60. The former (horizontal stretch) is explained as for Hadoop: the chunks are distributed more quickly when more nodes are involved. The latter stretch with the positive slope could be explained as follows. In the region of about 12 nodes, the network becomes saturated and is unable to pass more data in a time unit, while the mapping time is still proportionalto the chunk size: *T*_*comm*_≈const,*T*_*alignment*_∝*D*∝1/*N*⇒*T*_*alignment*_/*T*_*comm*_∝1/*N*; that is, there is a linear dependency in the selected coordinates, which can be observed in the plot. The transition area between the two modes is based on the fact that each hard disk drive on the local node can write data at a speed of about 1 Gbit/sec. Ten nodes will consume the data with a rate of 10 Gbit/sec, which is the limiting speed for the standard 10 Gbit Ethernet cable that is used to connect the cluster’s rack with the switch. The nodes are allocated on the same rack, which is the default SLURM behavior.

Scalability can be improved by overriding the default behavior of SLURM and allocating the nodes randomly from all available racks (‘HPC random’, open squares in Figure 5). Allocating the nodes on random racks allows more nodes to be engaged without saturating the network. For our cluster, we could go up to 30-35 nodes with perfect linear scaling. For the case where most resources are used (58 nodes), the deviation from a linear speedup is ≈7*%*; that is, 5.50 minutes against the ideal 5.14 (Table 3). The threshold number of nodes in this strategy (≈35) is caused because of saturation of the uplink cable at a throughput of 50 Gbit/s. The proposed HPC strategies that aim to get the maximum performance from the storage resources show that, even while being properly adjusted and tuned, the HPC approaches suffer from network saturation at a higher number of nodes. HDFS, on the other hand maintains data locality, reducing communication and data transfer and, hence, leads to better scalability. Although our investigation on scaling was based on our existing limited capacity, we expect the behavior to not significantly deviate from the scaling shown in Figure 5 [17,19].

**Table 3:** **Timings for mapping and the ratio** ***T***_***alignment***_ **/** ***T***_***comm***_ **for HPC and Hadoop I clusters for Dataset S as a function of the number of nodes involved**

Hadoop I			HPC random		
Number of nodes (cores)	Mapping *T*_*alignment*_	$\frac {T_{\textit {alignment}}}{T_{\textit {comm}}}$	Number of nodes (cores)	Mapping *T*_*alignment*_ time,minutes	$\frac {T_{\textit {alignment}}}{T_{\textit {comm}}}$
4(28)	293.5	1.71	4(64)	74.4	3.89
6(42)	189.8	1.62	10(160)	32.4	3.76
8(56)	136.0	1.62	14(224)	22.7	3.77
16(112)	70.3	1.48	18(288)	17.9	3.78
32(224)	39.3	1.66	22(352)	14.5	3.79
40(280)	32.5	1.65	26(416)	12.3	3.77
			30(480)	10.7	3.73
			34(544)	9.5	3.45
			38(608)	8.5	3.16
			42(672)	7.6	2.96
			46(736)	7.0	2.55
			50(800)	6.4	2.65
			54(864)	5.9	2.34
			58(928)	5.5	2.12

For the ‘HPC random’ approach, data chunks first have to be copied to the local node disks, and the alignments (SAM files) are copied back, while Hadoop keeps all of the data inside HDFS and, hence, does not need data staging. However, Hadoop needs to ingest the data to HDFS and preprocess the reads before the actual mapping stage so as to be able to operate in an MR manner, resulting in what we term ‘communication costs’. Note that each HPC node has 16 cores, while each Hadoop node has seven cores (the eighth core is dedicated to run the virtual machine).

### Usability

**Figure 6:**
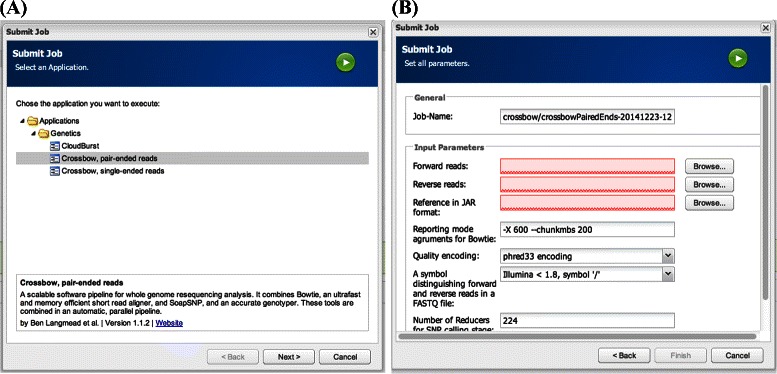
An example of a job setup view with the graphical Hadoop front-end Cloudgene, providing a smooth user experience, even for novice users.**A)** Pipeline selection, which in our case contains the Crossbow pipeline. **B)** Parameter setting for the job.

Hadoop presents a computing paradigm that is different from that which most users are accustomed to, and it requires an investment to learn how to use it efficiently. The Galaxy [13,42] platform is a good example of how to deliver bioinformatics pipelines on HPC and cloud resources. Galaxy provides a web-based graphical user interface (GUI) to bioinformatic programs, which simplifies the experience for the end user. CloudGene [43] is a similar project that uses a GUI to help lower the learning curve for Hadoop adopters. In addition, CloudGene is specifically targeted at users in the life sciences. It uses a light-weight and flexible web-based solution for both public and private clouds. In our study we integrated our Hadoop-pipeline in CloudGene and extended the platform with functions to import data from the central file system at UPPMAX into HDFS on the private cloud (see Figure 6). For our particular task, which is related to DNA sequencing, CloudGene makes it easy to select the data and parameters for pipeline execution. Most of the data management work is done automatically and the results can be downloaded to the client machine. The modular structure allows us to modify the source code to adapt it to the existing computing center architecture. For example, UPPMAX users can import their data from the sequencing platform directly to the Hadoop cluster by pressing a button and entering the credentials, while at the same time they can be sure that their sensitive data is kept private. It should be mentioned that although Crossbow has its own native user interface for running on Amazon EC2 [44], it does not support private clouds.

## Discussion

In this report we have described two approaches to the high-performance analysis of DNA sequencing data: one is based on regular HPC using a batch system and the other is based on Hadoop. To compare the two platforms, we developed highly optimized pipelines for both scenarios. We were able to show that for datasets of biologically reasonable size (i.e. 30*X* sequencing depth for human genome) Hadoop is comparable to HPC in terms of CPU-hours and is becoming an attractive option for bioinformatics data analysis from an e-infrastructure perspective. This conclusion is also supported by previously reported studies that have applied Hadoop to the analysis of RNA-seq [23] and de-novo sequence assembly [24] experiments. The main benefit of Hadoop is its almost linear scaling with the problem size. We were able to show that this also holds for analysis of NGS data. Our results further reveal that calculations on Hadoop with HDFS scale better than the network-attached parallel storage commonly used in HPC centers. For the already established HPC environment, MapReduce tasks can be submitted to multiple nodes within a cluster using MPI and the hybrid model. Projects such as MARIANE [45] report recent advances in this direction.

The amount of RAM installed on the nodes has a significant impact on Hadoop’s performance. Additional RAM benefits performance in at least two ways: firstly, it reduces the spilling from RAM to hard disk during the data aggregation phase between the Map and Reduce; and secondly, it allows (in our case) a speedup of 20% by enabling hyper-threading while preserving enough RAM for each logical core. Our results show that 4 GB of RAM per core is enough for Hadoop to efficiently analyze the results of *H.sapiens* sequencing experiments. On the other hand, if the hardware resources are modest, leaving insufficient memory for each core to keep the indexed genome in memory (i.e. for programs that do not implement more efficient shared memory strategies), it is more pragmatic to increase the amount of RAM available per core by leaving some cores idle.

Hadoop has so far seen relatively low adoption in bioinformatics. The primary reason for this is that the selection of bioinformatics software for Hadoop is still somewhat limited because most analysis tools are implemented as regular Linux applications rather than being written specifically for Hadoop. Although it is possible in some cases to wrap existing applications within adapter software (i.e. through purpose-built software, like Crossbow [22] adapts Bowtie [5], or through a generic adaptor like Hadoop Streaming library) regular Linux applications cannot generally be used directly on it. Two interesting frameworks to simplify scripting in Hadoop are BioPig [46] and SeqPig [47], which are both built on top of the Apache Pig framework and are capable of automatically distributing data and parallelizing tasks.

At the moment, however, Hadoop is incompatible with conventional HPC resources and requires specialized system administration; hence, adopting it into an existing HPC facility can be challenging. Furthermore, HDFS is not fully POSIX-compliant, which means it cannot be mounted as a regular Linux file system on computing nodes. In addition, the temporary resource allocation strategy used with HPC batch queues is a poor match for the local storage strategy used by HDFS. The fact that typical HPC nodes are usually equipped with relatively small amounts of local storage is a further sub-optimal design for HDFS storage nodes. There are currently efforts that try to circumvent these incompatibilities. For example, a recent study has provided Hadoop-on-Demand on HPC resources [48]. An alternative approach is to forego the HDFS and only use the Hadoop MapReduce component. Spark [49] is another emerging platform for distributed computing and it has recently been introduced in the bioinformatic domain [50]. It performs well for iterative tasks on computers with large amounts of RAM. ADAM [51] is a recent interesting NGS analysis framework and processing pipeline that is built on top of Spark. We intend to investigate ADAM’s performance in our future research.

In summary, the results of this work show that Hadoop, despite its limitations, enables highly scalable massively parallel analysis of DNA sequence data and can be justified from an economic e-infrastructure perspective for the data sizes that are common today. Given that the size of the datasets and the number of samples used in molecular bioscience are expected to increase over time, we envision that Hadoop will become increasingly popular in several types of bioinformatics applications.

## Availability and requirements

- **Project name:** Analysis pipelines to compare the Hadoop and HPC platforms,
- **Project home page:**https://github.com/raalesir/HPC_bash_alignhttps://github.com/raalesir/mr_python
- **Operating systems:** Unix
- **Programming language:** Python and Bash
- **Other requirements:** Unix
- **License:** GPLv3
- **Any restrictions to use by non-academics:** None

## Additional file

Additional file 1
**Supplemental information.** Description of computational facilities. 1. HPC: Multinode short-read mapping was performed on the Milou cluster (http://www.uppmax.uu.se/the-milou-cluster), equipped with dual eight-core Intel Xeon E5-2660 processors, (2.2 GHz, 2 MB L2 cache, 20 MB L3 cache), 128 GB of RAM, an Infiniband node-to-node network connection, and a 10Gbit/s uplink. 2. Storage: Gulo (http://www.uppmax.uu.se/gulo) is a custom built Lustre 2.4 system using eight HP nodes with MDS600 storage boxes and an additional node for metadata handling. In total, it provides roughly 1 PB of storage and is accessed with Lustre’s own protocol. It supports data striping over multiple nodes and disk targets, and can give a theoretical single file read performance of up to 80 Gbits per second. 3. The Hadoop I cluster was deployed using OpenNebula. Each physical node was equipped with two quad-core Intel Xeon 5520 processors (clock frequency of 2.26 GHz; 1 MB L2 cache, 8 MB L3 cache), 72 GB of RAM, one 2 TB SATA disk, and Gigabit Ethernet. The hyper-threading function was used to address 112 logical cores instead of 56 physical for the Hadoop II cluster.

## Abbreviations

HPC: High-performance computing
NGS: Next-generation sequencing
Gb: Gigabases: GB: Gigabytes
TB: Terabytes
MPI: Message-passing interface: MR: MapReduce
HDFS: Hadoop distributed file system
RAM: Random-access memory
CPU: Central processing unit
SNP: Single nucleotide polymorphism
NAS: Network-attached storage
SAN: Storage area network
SRA: Short read archive
NFS: Network file system
SLURM: Simple linux utility for resource management: GUI: Graphical user interface
