# Erratum to: A quantitative assessment of the Hadoop framework for analyzing massively parallel DNA sequencing data

**DOI:** 10.1186/s13742-015-0100-7

**Published:** 2015-12-09

**Authors:** Alexey Siretskiy, Tore Sundqvist, Mikhail Voznesenskiy, Ola Spjuth

**Affiliations:** 1 Department of Information Technology, Uppsala University, P.O. Box 337, Uppsala, SE-75105 Sweden; 2 Department of Physical Chemistry, Institute of Chemistry, St-Petersburg State University, Saint-Petersburg, Russia; 3 Department of Pharmaceutical Biosciences and Science for Life Laboratory, Uppsala University, P.O. Box 541, Uppsala, SE-75124 Sweden

## Erratum

The original version of this article [1] unfortunately contained a publisher error in Fig. 4. The figure was incorrectly captured as a duplicate of Fig. 5. The correct Fig. 4 has been published in this Erratum. See Fig. 1.

**Fig. 1:**
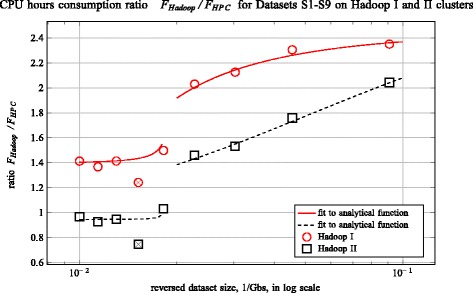
The ratio of the *F*_*Hadoop*_/*F*_*HPC*_ as a function of the reciprocal dataset size in Gb. The pipelines were run on the Hadoop I and II clusters, as well as a 16 core HPC node. The analytical curve *f*(*x*) = (*a*_1_*x* + *b*_1_)/(*a*_2_*x* + *b*_2_) was used to fit the data for the stretches of linear scaling of calculation time on the HPC platform. The outliers are marked with crossed symbols
