# Supplementary material for: A quantitative assessment of the Hadoop framework for analyzing massively parallel DNA sequencing data
Source: Gigascience. 2015 Jun 4;4:26. doi: 10.1186/s13742-015-0058-5 (PMC4455317; doi:10.1186/s13742-015-0058-5)
Supplement: Additional file 1 — Supplemental information. Description of computational facilities. 1. HPC: Multinode short-read mapping was performed on the Milou cluster (http://www.uppmax.uu.se/the-milou-cluster), equipped with dual eight-core Intel Xeon E5-2660 processors, (2.2 GHz, 2 MB L2 cache, 20 MB L3 cache), 128 GB of RAM, an Infiniband node-to-node network connection, and a 10Gbit/s uplink. 2. Storage: Gulo (http://www.uppmax.uu.se/gulo) is a custom built Lustre 2.4 system using eight HP nodes with MDS600 storage boxes and an additional node for metadata handling. In total, it provides roughly 1 PB of storage and is accessed with Lustre’s own protocol. It supports data striping over multiple nodes and disk targets, and can give a theoretical single file read performance of up to 80 Gbits per second. 3. The Hadoop I cluster was deployed using OpenNebula. Each physical node was equipped with two quad-core Intel Xeon 5520 processors (clock frequency of 2.26 GHz; 1 MB L2 cache, 8 MB L3 cache), 72 GB of RAM, one 2 TB SATA disk, and Gigabit Ethernet. The hyper-threading function was used to address 112 logical cores instead of 56 physical for the Hadoop II cluster. [file 13742_2015_58_MOESM1_ESM.pdf]

Short supplementary scripts

BASH script for collecting statistics for `gz` to `bz` re-archiving using GNU parallel tool

```
for file in `ls ${dir1}/*.gz`;do
        for ((i=0; i<3; i++));do
                /usr/bin/time -o ${timeFile} -p -a -f "%e sec 
                    converting gz to bz for  $file " \
                sh -c "ls  $file | parallel 'zcat {} | pbzip2 >${dir2
                    }/{/.}.bz2'"
        done
done
```

BASH script for collecting statistics for splitting `gzip` archives using GNU parallel and `pigz` tools

```
for file in `ls -v ${dir1}/*.gz`;do
        for ((i=0; i<2; i++));do
                d=${dir1}/${dir2}/`basename $file`;     mkdir -p $d
                time pigz -dck   $file |parallel --no-notice --pipe -N
                     10000000  "gzip  >$d/{#}.gz"  >>${timeFile}
        done
done
```
